# Key Interleukins in Inflammatory Bowel Disease—A Review of Recent Studies

**DOI:** 10.3390/ijms26010121

**Published:** 2024-12-26

**Authors:** David Aebisher, Dorota Bartusik-Aebisher, Agnieszka Przygórzewska, Piotr Oleś, Paweł Woźnicki, Aleksandra Kawczyk-Krupka

**Affiliations:** 1 Department of Photomedicine and Physical Chemistry, Medical College of The Rzeszów University, 35-310 Rzeszów, Poland; 2 Department of Biochemistry and General Chemistry, Medical College of The Rzeszów University, 35-310 Rzeszów, Poland; dbartusikaebisher@ur.edu.pl; 3 English Division Science Club, Medical College of The Rzeszów University, 35-310 Rzeszów, Poland; ap117623@stud.ur.edu.pl (A.P.); pw118616@stud.ur.edu.pl (P.W.); 4 Department of Internal Medicine, Angiology and Physical Medicine, Center for Laser Diagnostics and Therapy, Medical University of Silesia in Katowice, Batorego 15 Street, 41-902 Bytom, Poland; piotroles@o2.pl

**Keywords:** interleukins, inflammatory bowel disease, recent studies, ulcerative colitis, Crohn’s disease, interleukin 1β, interleukin 6, interleukin 10, interleukin 17, interleukin 22, interleukin 12, interleukin 23, interleukin 33

## Abstract

Inflammatory bowel disease (IBD) is an immune disorder of the gastrointestinal tract with a complex aetiopathogenesis, whose development is influenced by many factors. The prevalence of IBD is increasing worldwide, in both industrialized and developing countries, making IBD a global health problem that seriously affects quality of life. In 2019, there were approximately 4.9 million cases of IBD worldwide. Such a large number of patients entails significant healthcare costs. In the treatment of patients with IBD, the current therapeutic target is mucosal healing, as intestinal inflammation often persists despite resolution of abdominal symptoms. Treatment strategies include amino salicylates, corticosteroids, immunosuppressants, and biologic therapies that focus on reducing intestinal mucosal inflammation, inducing and prolonging disease remission, and treating complications. The American College of Gastroenterology (ACG) guidelines also indicate that nutritional therapies may be considered in addition to other therapies. However, current therapeutic approaches are not fully effective and are associated with various limitations, such as drug resistance, variable efficacy, and side effects. As the chronic inflammation that accompanies IBD is characterized by infiltration of a variety of immune cells and increased expression of a number of pro-inflammatory cytokines, including IL-6, TNF-α, IL-12, IL-23 and IFN-γ, new therapeutic approaches are mainly targeting immune pathways. Interleukins are one of the molecular targets in IBD therapy. Interleukins and related cytokines serve as a means of communication for innate and adaptive immune cells, as well as nonimmune cells and tissues. These cytokines play an important role in the pathogenesis and course of IBD, making them promising targets for current and future therapies. In our work, we review scientific studies published between January 2022 and November 2024 describing the most important interleukins involved in the pathogenesis of IBD. Some of the papers present new data on the precise role that individual interleukins play in IBD. New clinical data have also been provided, particularly on blocking interleukin 23 and interleukin 1beta. In addition, several new approaches to the use of different interleukins in the treatment of IBD have been described in recent years.

## 1. Introduction

**Figure 1:**
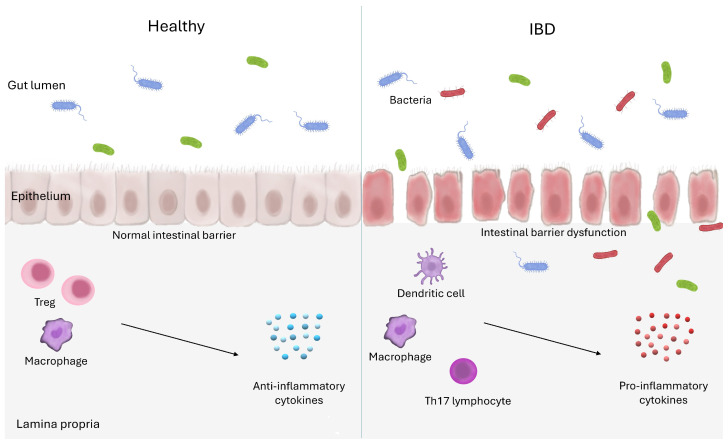
The figure shows a cross section of a healthy bowel and a bowel affected by inflammatory bowel disease (IBD). The figure shows the differences in intestinal barrier structure, epithelial permeability, and immune cell activity.

Inflammatory bowel disease (IBD) is an immune disorder of the gastrointestinal tract with a complex aetiopathogenesis, the development of which is influenced by many factors, including genetic history and environmental factors such as stress, sleep patterns, antibiotic use, hygiene, diet, and smoking [1,2,3,4,5]. The prevalence of IBD is increasing worldwide, both in industrialized and developing countries, making IBD a global health problem that seriously affects patient quality of life [1,6,7]. In 2019, there were approximately 4.9 million cases of IBD worldwide [8]. Such a large number of patients incurs significant health care costs. These estimates do not take into account the “real” price of IBD, which can hinder career aspirations, instill social stigma, and diminish patient quality of life [9]. Moreover, IBD can lead to the development of serious health consequences, such as colorectal cancer [10]. In a healthy gut, the intestinal barrier is integral, providing effective protection against the penetration of bacteria, toxins, and other antigens into the mucosa. The immune system in a healthy gut operates in a state of homeostasis, allowing tolerance to food antigens and elements of the intestinal microbiota, while providing effective defense against pathogens. Regulatory T cells (Treg) and macrophages with an M2 phenotype play an important role in regulatory processes, supporting the production of anti-inflammatory cytokines [11,12]. This coordinated action of the physical barriers, the microbiota, and the immune system ensures that the gut functions properly and protects the body from threats. In IBD, epithelial barrier function is disrupted, leading to increased intestinal permeability [13]. As a result of damage to the intercellular junctions, spaces are created between epithelial cells, allowing bacteria and their products to enter the intestinal mucosa and significantly alter the intestinal microbiota, thus exacerbating the degree of dysbiosis [14]. There, bacteria interact with immune cells, initiating an inflammatory response. Inflammation further damages the epithelial layer, increasing intestinal permeability and allows other pathogens to penetrate, exacerbating the inflammatory response. The intestinal mucosa in people with IBD is characterized by infiltration of numerous inflammatory cells, such as dendritic cells, macrophages mainly of the M1 phenotype, Th17 lymphocytes, and other immune effector cells. These cells produce significant amounts of pro-inflammatory cytokines and chemokines, which, together with other inflammatory mediators, cause tissue damage and perpetuate chronic inflammation [15]. The dominance of pro-inflammatory cytokines in the mucosa not only exacerbates tissue-destructive processes, but also impairs the regenerative capacity of the epithelium, resulting in further disruption of the intestinal barrier. This creates a vicious circle in which increased inflammation leads to further damage to the intestinal barrier, which in turn promotes an escalation of inflammation. The basics of IBD pathomechanisms are shown in Figure 1.

Inflammatory bowel disease mainly consists of two distinct clinical phenotypes: ulcerative colitis (UC) and Crohn’s disease (CD) [1]. Ulcerative colitis is generally associated with continuous colitis, starting in the rectum and spreading to the proximal segments of the colon, affecting mainly the inner layer of the colonic epithelium. It affects mainly adults aged 30–40 years, although its incidence among children and adolescents is increasing. Ulcerative colitis can be divided according to the Montreal classification by the area of inflammation involved: E1 (proctitis): Lesions limited to the rectum, E2 (left-sided colitis): Lesions involving the left side of the colon up to the splenic fold, E3 (extensive colitis): Extensive inflammation involving the colon extends beyond the splenic fold [16]. Crohn’s disease is a chronic inflammatory disease of the gastrointestinal tract that can lead to progressive intestinal damage and disability. Crohn’s disease can affect any part of the digestive tract from the mouth to the anus. Typical are segmental inflammatory lesions, punctuated by healthy sections [17,18]. Due to the lack of specific diagnostic markers, the diagnosis of IBD still requires a gold standard based on a combination of clinical symptoms, imaging, laboratory, and endoscopic findings. The identification of characteristic ileocolonoscopy and histological findings remains the cornerstone of IBD diagnosis, but a complete evaluation includes laboratory abnormality detection, including micronutrient deficiencies, cross-sectional imaging to determine the extent, severity, and complications of IBD, and psychosocial evaluation [19]. In the treatment of patients with IBD, the current therapeutic target is mucosal healing, as intestinal inflammation often persists despite resolution of abdominal symptoms. The American College of Gastroenterology (ACG) guidelines report that endoscopic scores have been developed that are reliable for measuring mucosal healing and can be used to monitor response to treatment [20,21]. Treatment strategies focus on reducing intestinal mucosal inflammation, inducing and prolonging disease remission, and treating complications [22]. Currently, pharmacological intervention is important in the treatment of IBD. Medications include aminosalicylates, corticosteroids, immunomodulators, biologics, and oral small molecules [23]. Aminosalicylates used in IBD include the traditional sulphasalazine (SASP), and other types of drugs containing 5-aminosalicylic acid (5-ASA). Oral 5-ASA has better efficacy in the treatment of UC than placebo [24]. A meta-analysis reported efficacy of topical 5-ASA in preventing recurrence of UC [25]. The therapeutic efficacy of aminosalicylic acid preparations in CD remains controversial, and the available data is disparate [26,27]. Side effects associated with 5-ASA include bloating, nausea, abdominal pain, diarrhea, and headaches that are generally mild. In contrast, the side effects of SASP, such as infertility, hemolytic anemia, photosensitization, and granulocytosis, are significantly greater than those of 5-ASA [28]. Oral corticosteroids can be effective in inducing remission in exacerbations but have no demonstrated efficacy in maintaining remission in IBD and should not be used for this purpose [23,29]. Furthermore, systemic oral glucocorticosteroids can cause numerous side effects, such as opportunistic infections, hypertension, venous thromboembolism, osteoporosis, and many more [30]. Immunomodulators are important for patients with IBD and include thiopurines, methotrexate, calcineurin inhibitors, and Janus kinase inhibitors [31]. Thiopurines and methotrexate are mainly effective in CD, with thiopurines more burdened by side effects. Calcineurin inhibitors, especially tacrolimus, have good results in refractory UC, but their safety is limited due to severe side effects [23,32,33]. Biological drugs used in IBD include inhibitors of pro-inflammatory cytokines and integrin antagonists. They are very effective and selective in IBD, but their high cost and problems with non-response or loss of efficacy pose clinical challenges [23]. Anti-TNF therapy is effective in IBD, but up to 40% of patients do not respond to treatment and up to 46% lose response after 1 year [34]. Anti-integrin therapy, which blocks the action of integrins on the surface of leukocytes and endothelial CAM, thus inhibiting the interaction of leukocytes with the intestinal mucosa, is achieving promising results in the treatment of IBD [35]. Many studies have suggested that IL-12/23 and IL-23 antagonists are potential therapeutic options for the treatment of IBD. Experts have recommended IL-12/23 and IL-23 antagonists as first- or second-line therapies due to their efficacy in biologically naïve and treatment-experienced patients [36]. Orally absorbed small molecules are of great interest to researchers because of the convenience of oral administration [23]. Both JAK inhibitors and S1P modulators are modern therapeutic options in IBD. They show significant efficacy, especially in patients refractory to other treatments, but their long-term safety requires further study [37,38,39]. For a precise overview and comparison of therapeutic options in the treatment of IBD, we recommend the work of Cai et al., and Jeong et al. [23,31]. American College of Gastroenterology guidelines suggest that dietary therapies may be effective in some patients. However, these benefits are not sustainable, with symptoms and active inflammation returning after resumption of an unrestricted diet. Therefore, dietary therapies may be considered as an adjunct to other therapies [20,21] As the chronic inflammation that accompanies IBD is characterized by infiltration of a variety of immune cells and increased expression of a number of pro-inflammatory cytokines, including IL-6, TNF-α, IL-12, IL-23, and IFN-γ, new therapeutic approaches are mainly targeting immune pathways [40]. One of the molecular targets in IBD therapy are interleukins. Interleukins and related cytokines serve as a means of communication for innate and adaptive immune cells, as well as non-immune cells and tissues [41]. These cytokines play an important role in the pathogenesis and course of IBD, making them useful for treatment and promising targets for future therapies. Research into the precise elucidation of the role of interleukins in IBD is still ongoing. In our paper, we summarize the results of recent studies published in the last 2 years (January 2022 to November 2024) focusing on the most important interleukins in IBD. We summarize newly described mechanisms, clinical data, and novel therapeutic approaches based on these cytokines.

## 2. Interleukins

### 2.1. Interleukin 1β

Interleukin-1β (IL-1β) is a potent pro-inflammatory cytokine that is crucial for the host defense response to infection and injury [42]. This cytokine plays a critical role in the development of many autoimmune diseases [43]. The balance between IL-1β and its endogenous inhibitor, IL-1Ra, plays a key role in both the initiation and regulation of inflammation [44]. IL-1β is an important mediator of inflammation and tissue damage in IBD as well. IL-1β is mainly found in a secretory form, almost always released by peripheral blood monocytes and cells isolated from the inflamed gastrointestinal mucosa of patients with IBD [45]. Although the involvement of IL-1β in the pathogenesis of IBD is well documented, the exact role of IL-1β remains unclear [46]. Recent studies have focused on the use of IL-1β as a novel therapeutic target in IBD. Despite the fact that IL-1β is considered to be as important a cytokine as TNF-α at the onset of IBD, there are currently few specific antibodies against IL-1β tested in IBD with the exception of canakinumab [47]. Shaul et al. evaluated the efficacy and safety of canakinumab in children with very early-onset inflammatory bowel disease (VEO-IBD) with an autoinflammatory phenotype. Nineteen patients were included in the study. At baseline, 37% of patients were not receiving biologics, and canakinumab was used in dual therapy in 74% of patients. Clinical response was achieved in 89% of patients with statistically significant improvement. Clinical remission was achieved in 32% of patients. Significant improvements in clinical symptoms and biochemical markers of the disease were observed, and the number of hospitalizations and the lengths of hospital stays were also reduced [48]. Another molecule that blocks IL-1 β-transmission is anakinra, which inhibits the effects of both IL-1 β and IL-1α by blocking the IL-1 receptor. In 2023, Truyens et al. presented a case report of a UC patient successfully treated with this pharmaceutical [49]. In recent years, two novel approaches to treating IBD based on targeting IL-1β have also been presented (Table 1). Cai et al. described a molecule, 10v, providing a novel approach to the treatment of UC through two mechanisms: blocking IL-1β signaling via the NLRP3 and AIM-2 inflamasome and reducing STAT1 and STAT5 expression in the JAK/STAT pathway. In a mouse model of UC, 10v showed better efficacy than the clinically used IBD treatment sulfasalazine [50]. Zhu et al. showed that reducing IL-1β production and selectively inhibiting the NLRP3 inflamasome through activation of the estrogen receptor β (Erβ) significantly reduces the severity of colitis symptoms in a mouse model. Use of this receptor agonist resulted in reduced weight loss, intestinal shortening, histological damage, and MPO activation in intestinal tissue, and the treatment effects were comparable to the standard treatment of IBD, 5-aminosalicylic acid [51].

### 2.2. Interleukin 6

**Figure 2:**
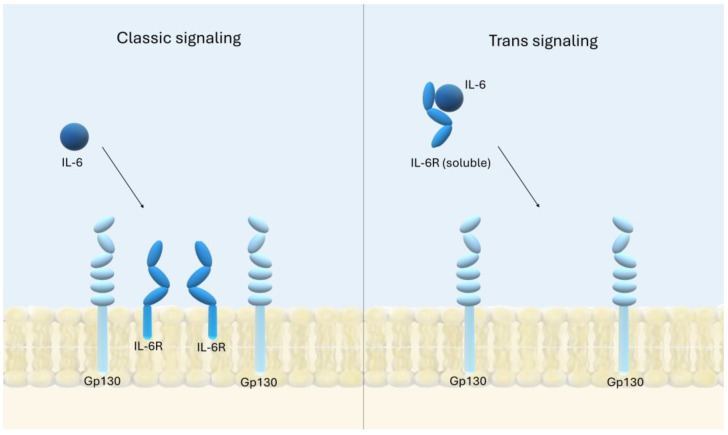
IL-6 signaling occurs through two mechanisms: classical and trans. Classical signaling occurs via the membrane-bound IL-6R receptor and affects a limited number of cells that express the membrane-anchored form of IL-6R and gp130. Trans signaling is mediated by the soluble IL-6R receptor and affects cells that do not have membrane expression of IL-6R but do have gp130. By affecting all gp130-expressing cells, the mechanism is almost universal. Trans-signaling is thought to be the reason for the chronic inflammatory effect of IL-6.

Interleukin 6 (IL-6) is a long-chain, four-helix signaling protein that acts through its cytokine-specific receptors and the ubiquitous trans-membrane receptor gp130 (CD130) to transmit a single-pass signal [52]. It was originally identified as a regulator of B cell differentiation in 1986 [53]. IL-6 plays a critical role in the development of IBD by affecting immune cells [54]. In IBD, increased levels of this interleukin are observed in patients [55]. The main sources of IL-6 in IBD are intestinal epithelial cells, mononuclear lamina propria phagocytes, mesenchymal cells, and T lymphocytes [56]. IL-6 contributes to damage of the intestinal epithelial barrier during inflammation by increasing its permeability through increased expression of claudin-2, a tight junction protein responsible for permeability to small cations. These effects are well documented in Caco-2 cell cultures and in patients with CD and UC. IL-6 also decreases occludin and claudin-1 levels, further increasing intestinal permeability [57]. Furthermore, IL-6 regulates the immune response in the gut by activating the NFκB pathway, which increases ICAM-1 expression, facilitating neutrophil-epithelium interaction during inflammation [58]. IL-6 signaling occurs through two mechanisms: classical and trans. Classical signaling occurs via the membrane-bound IL-6R receptor and affects a limited number of cells that express the membrane-anchored form of IL-6R and gp130 [57]. It has been shown to be involved in TLR activation of pro-inflammatory signaling pathways [52,59]. Trans signaling is mediated by the soluble IL-6R receptor and affects cells that do not have membrane expression of IL-6R but do have gp130. By affecting all gp130-expressing cells, the mechanism is almost universal. Trans-signaling is thought to be the reason for the chronic inflammatory effect of IL-6 [52,57]. The mechanisms of IL-6 signaling are shown in Figure 2.

Despite the well-established role of IL-6 in the development of IBD, global IL-6 blockade has been found to cause intolerable side effects (e.g., intestinal perforations) [60,61].

Recent work describes attempts to block IL-6 and determine its function as a marker of disease progression. Selective inhibition of IL-6 trans-signaling may provide clinical benefit with fewer side effects than in the case of global IL-6 blockade. Recently, innovative bi-specific cytokine inhibitors have been developed by Gesiorowski et al. that combine the properties of blocking IL-6 trans signaling and TNF or IL-12/23 signaling. The developed inhibitors have shown high affinity for IL-6/sIL-6R and TNF or IL-12/23 complexes. One of them, cs130-TNFVHHFc, effectively inhibited TNFα-induced apoptosis of L929 cells, suggesting that it may act similarly to currently used anti-TNF drugs, but with the added benefit of blocking IL-6 (Table 2). These inhibitors were also effective in inhibiting STAT3 phosphorylation, indicating that they block key inflammatory pathways in IBD [62]. Furthermore, significantly higher levels of IL-6 were found in patients with low and excessive body fat compared to normal fat subjects. Godala et al. showed that measurements of IL-6 and IL-1β levels can provide additional information about the nutritional status of IBD patients, especially in the context of body fat and muscle mass [63].

### 2.3. Interleukin 10

**Figure 3:**
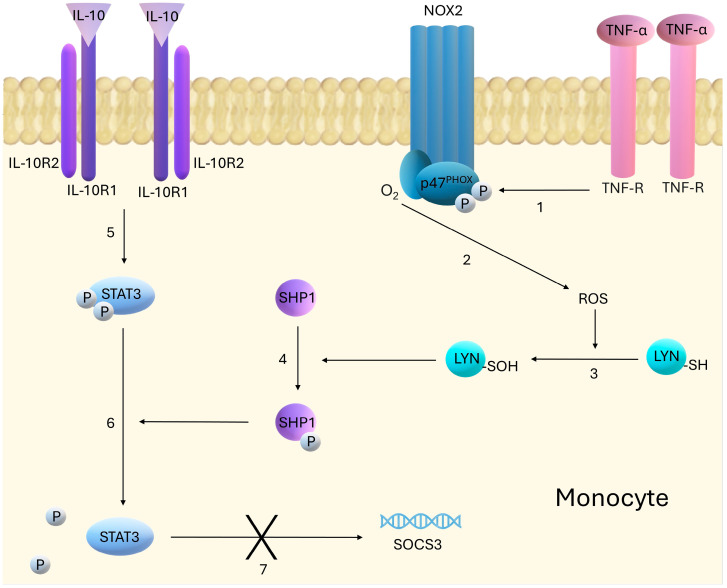
The figure shows the NOX2-ROS-Lyn-SHP-1 pathway in monocytes through which TNFα inhibits STAT3 phosphorylation induced by IL-10. 1. TNF-α, acting through its receptors (TNF-R), phosphorylates the p47PHOX subunit of NOX2, leading to activation of the complex. 2. The activated NOX2 complex generates reactive oxygen species (ROS) from NADPH and molecular oxygen (O_2_). 3. ROS activate Lyn kinase, oxidizing thiol groups of cysteines (-SH) to sulfenic groups (-SOH). 4. Activated Lyn kinase phosphorylates tyrosine phosphatase (SHP-1), which is crucial for its activity. 5. IL-10 acts on monocytes by activating a receptor composed of two subunits IL-10R1 and IL-10R2. Activation of this receptor leads to STAT3 phosphorylation. 6. Active SHP-1de-phosphorylates STAT3, leading to its inactivation. 7. Disruption of STAT3 phosphorylation leads to inhibition of SOCS3 (suppressor of cytokine signaling 3) expression, which is the major anti-inflammatory gene activated by STAT3.

Interleukin-10 (IL-10) is an immunoregulatory cytokine that is secreted by a wide range of immune cells, including macrophages, dendritic cells, and T lymphocytes [64]. This cytokine was discovered in 1989 as a Th2 cell-produced factor that inhibits Th1 cell function [65]. Since then, the protective functions of this IL have been established through the regulation of overexpressed immune responses and autoimmune pathologies [66]. IL-10 is highly relevant to IBD, as evidenced by the development of spontaneous intestinal inflammation in both IL-10−/− and IL-10Rβ−/− mice [67,68]. In humans, polymorphisms of IL-10 and its receptors have been found to be correlated with very early onset of colitis [69]. In relation to the importance of IL-10 in the development of IBD, a number of studies have been undertaken to date examining the use of this IL in the treatment of IBD [70]. Recent research has focused on elucidating the role of IL-10 in the pathogenesis of IBD and attempts to use it in therapy (Table 3). Mutations in genes encoding IL-10 or its receptors are known to cause severe forms of IBD in infants, leading to the development of inflammation that is often resistant to treatment. Griffin et al. demonstrated that neutralizing autoantibodies against IL-10 can induce IBD, resembling IL-10-related innate immune defects [71]. An increase in IL-10 levels is therefore desirable in IBD. An increase in IL-10 expression has been successfully achieved in recent studies by, among others, mesenchymal stem cells, microbiota or, for the first time studied in this context, pharmacological potassium channel KCa3.1 blockade [72,73,74]. Currently, after promising results in a mouse model, the AMT-1 molecule (#NCT04583358), which acts by concentrating biologically active IL-10 on the intestinal lamina propria, is in phase 2 clinical trials [75,76]. However, achieving efficacy of IL-10 therapy in the clinic may face difficulties. Recent work by Ben-Khemis et al. partially explains the mechanism by which IL-10 may lose efficacy. They showed that through the NOX2-ROS-Lyn-SHP-1 pathway, TNFα inhibits STAT3 phosphorylation induced by IL-10. This mechanism is illustrated in Figure 3. They found that SHP-1 inhibitors (such as NSC-87877) can block this mechanism, restoring STAT3 phosphorylation and protection against inflammation. The mechanism suggests that combining anti-TNFα therapy with SHP-1/2 inhibitors could enhance the efficacy of IL-10 therapy in the treatment of inflammatory diseases [77]. Furthermore, patients with autoantibodies to IL-10 may benefit from therapies that target the removal of B cells that produce these autoantibodies [71].

### 2.4. Interleukin 17

The interleukin 17 (IL-17) family of cytokines consists of six different cytokines (IL-17A to IL-17F) and five IL-17 receptors (IL-17RA to IL-17RE) [78]. IL-17 is the main cytokine secreted by Th17 lymphocytes [79]. The role of this cytokine in IBD is complex and not fully understood, and the degree of involvement of specific subtypes of this cytokine varies in UC and CD [78]. Despite the apparent wealth of data from mouse models, human descriptive studies and genetics, the effects of anti-IL-17 treatment are disappointing [80]. Two IL-17-blocking agents have been evaluated in patients with moderate to severe Crohn’s disease: secukinumab, which targets IL-17 A, and brodalumab, which blocks a key subunit of the IL-17 receptor, IL-17RA. Although the study designs for secukinumab and brodalumab differed, both showed worse outcomes in Crohn’s patients treated with IL-17 blockade than in the placebo group [81,82]. A possible reason for the disappointing results of these studies is that, in addition to its ability to induce mucosal inflammation, IL-17A may also contribute to the regeneration and repair of the intestinal mucosa after inflammation has subsided [83]. Recent studies clarify the role this cytokine plays in IBD (Table 4). Cai et al. showed that IL-17B, IL-17E, and IL-17RB are associated with UC, while IL-17C and IL-17RC are associated with CD [84]. IL-17A and IL-17F, through various signaling pathways such as NF-Kb, C/EBP, and MAPK, have been shown to activate the production of numerous inflammatory mediators (TNFα, IL-1β, IL-6, G-CSF, GM-CSF), chemokines (CXCL1, CXCL5, CCL2 and CCL7), and antimicrobial peptides, thereby enhancing the mucosal immune response in IBD [85].

### 2.5. Interleukin 22

Interleukin 22 (IL-22) is an α-helical cytokine belonging to the IL-10 family [86]. This molecule is a relatively newly characterized cytokine described in 2000 [87,88]. It is mainly produced by T helper cells (Th1, Th17 and Th22 cells), innate lymphoid group 3 cells (ILC3s), and neutrophils [89]. Clinical samples from patients with active UC and CD showed increased levels of IL-22 and an abundance of IL-22-expressing cells [90]. The biological effects of IL-22 are mediated by its binding to class 2 cytokine receptors, which consist of heterodimeric complexes comprising IL-10R2 and IL-22R1 [86]. The IL-22 receptor is expressed exclusively on cells of non-hematopoietic origin, such as IECs, and this receptor engagement results in cell proliferation and tissue repair following injury [89]. In addition to the IL-22 receptor complex, there is a soluble single-chain IL-22 receptor on the cell surface referred to as IL-22 binding protein (IL-22BP) or IL-22RA2. IL-22BP has the ability to antagonize IL-22 by occupying the IL-22 binding site to IL-22R1. Consequently, direct binding of IL-22 to IL-22BP inhibits the action of IL-22 [86]. The JAK1/TYK2-STAT pathway serves as a major signaling pathway for IL-22, with STAT3 playing a key role in this process [91]. IL-22 has two main functions: It strengthens the intestinal barrier by promoting tight gut connections and increases antimicrobial peptides and mucins to enhance mucosal defenses. The role of IL-22 in IBD is controversial. Most studies have shown that L-22 plays a protective role in the acute phase of tissue injury due to its promotion of epithelial cell proliferation and production of antimicrobial compounds. On the other hand, IL-22 may also induce inflammation when chronically overexpressed, resulting in increased production of inflammatory chemokines. Furthermore, if IL-22 signaling is not properly controlled, it can also induce malignancy [89].

Recently, research involving IL-22 has mainly focused on increasing knowledge of its mechanisms of action in IBD. Several novel protective functions of IL-22 in IBD have been described (Table 5). He et al. demonstrated a key role for IL-22 in the differentiation of Paneth cells [92]. Chen et al. demonstrated the significant involvement of IL-22 in the function of mesenchymal stem cells derived from Peyer’s tufts (MSCsPP). These cells exhibited immunomodulatory abilities, suppressed T-cell proliferation and production of pro-inflammatory cytokines such as TNF-α, IFN-γ, and IL-17, and, in a mouse model, alleviated the symptoms of IBD, as manifested by faster weight recovery, longer bowel length, and improved histological indices of tissue damage [93]. Zhu et al. showed that treatment of a mouse model of colitis caused by *Citrobacter rodentium,* IL-22.Fc improved survival by reducing the severity of colitis and decreasing weight loss. Furthermore, IL-22.Fc restored homeostatic levels of proteins associated with Na^+^/Cl^−^ transport and intestinal barrier function (e.g., SLC26A3, CAR4) and prevented the accumulation of undifferentiated epithelial cells, allowing them to mature into functional colonocytes and cup cells [94]. Two papers provide evidence for the involvement of other molecules in reducing the protective function of IL-22. Ninnemann et al. described the involvement of TNF in reducing the protective function of IL-22. In their novel work, they found that TNF interferes with the tissue-repair program by inducing a soluble natural IL-22 antagonist (IL-22Ra2; IL-22BP) in the colon and abolishes IL-22/STAT3-mediated mucosal repair during colitis. Furthermore, membrane-bound TNF expressed by T cells fixes colonic inflammation, while soluble TNF produced by IECs induces IL-22BP expression in colonic dendritic cells and suppresses IL-22-induced restoration of colonic epithelial function. TNF induces IL-22BP expression in human monocyte-derived DCs, and IL22-BP levels correlate with TNF in the serum of IBD patients [89]. The work of Fantou et al. provides novel information on the biology of IL-22BP and its role in the pathophysiology of CD. Higher levels of IL-22BP were detected in the ileum than in the colon in both CD patients and controls. The IL-22/IL-22BP ratio, reflecting IL-22 activity, was higher in the colon of CD patients, suggesting greater bioavailability of IL-22 in this location. Furthermore, Fantou et al. found that the main sources of IL-22 BP were eosinophils and mononuclear phagocytes, and that active smokers had higher levels of IL-22BP in the colon [95]. Recent studies have also increased knowledge of the harmful effects of IL-22 in IBD. Breugelmans et al. described a novel mechanism in which IL-22 induces intestinal barrier dysfunction via the MUC13 protein [96]. Pavlidis et al. demonstrated that IL-22 regulates neutrophil recruitment in UC and is associated with resistance to ustekinumab treatment [97]. Pravoverov et al. showed that IL-22 increases the expression of oncogenic microtubule-associated serine/threonine kinase (MASTL). It is likely that IL-22 enhances MASTL protein stability by promoting its association with carbonic anhydrase IX (CAIX) potentially through AKT signaling to promote cell survival and proliferation [98]. Kuchař et al. made a promising attempt to modulate the IL-22/IL-22R1 pathway to improve the course of IBD. They demonstrated the efficacy of using small antagonistic binding proteins for this purpose. Administration of one of these proteins, ABR167, reduced the clinical and histological manifestations of colitis, such as shortened bowel length and changes in epithelial structure and inflammatory infiltration in a mouse model of IBD [99].

### 2.6. Interleukins 23 and 12

**Figure 4:**
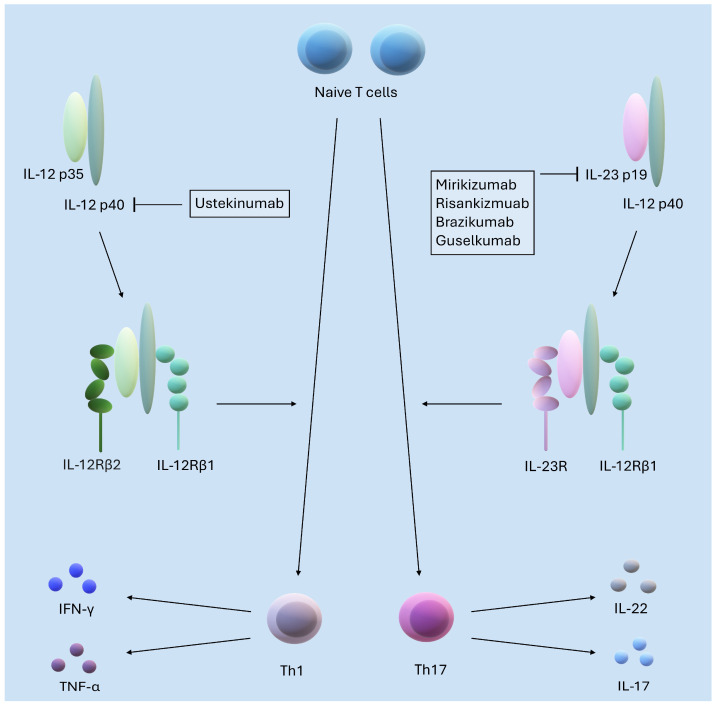
The figure shows IL-12, which induces polarization of naive T cells towards Th1 cells, which then produce pro-inflammatory cytokines such as IFN-γ and TNFα. IL-23, on the other hand, is involved in the differentiation of naive T lymphocytes towards Th17 lymphocytes, which increase the secretion of other inflammatory cytokines such as IL-17 and IL-22. IL-12 and IL-23 are heterodimeric cytokines that share the IL-12p40 subunit. The IL-12p35 subunit binds to the IL-12Rβ2 receptor, while IL-23p19 binds to the IL-23R receptor, which induces structural changes that facilitate the association of the IL-12p40 subunit with the IL-12Rβ1 chain. The monoclonal antibody ustekinumab binds to the IL-12p40 subunit, blocking both IL-12 and IL-23 cytokine signaling, while antibodies such as mirikizumab, risankizumab, brazikumab, and guselkumab target the IL-23p19 subunit.

Interleukin 23 (IL-23) and interleukin 12 (IL-12) are heterodimeric cytokines composed of the common IL-12p40 subunit and the IL-23p19 and IL-12p35 subunits, respectively [100]. These interleukins are mainly produced by inflammatory marrow cells, including activated antigen-presenting cells [101]. IL-12 is involved in the initiation of intestinal inflammation caused by epithelial barrier disruption and collaborates with IL-23 to maintain chronicity, with IL-12 being more prominent in earlier stages and IL-23 in later stages [102]. IL-23 is also involved in the differentiation of naive T17 helper cells, which increase the secretion of other inflammatory cytokines such as IL-17 and IL-22 [103,104]. On the other hand, IL-12 has been found to induce Th1 polarization, which is further associated with the production of IFN-γ and TNFα and the recruitment of macrophages, NK cells, and CD8+ T cells [105]. The above information is illustrated in Figure 4.

Therefore, IL-12 and IL-23 play a key role in maintaining inflammation in IBD [103]. Despite ongoing debate and research into the precise signaling pathways of both of these interleukins, their role in the pathogenesis in IBD is well established [104,106,107]. Recent work advances current knowledge of the function of these cytokines in IBD, examines the potential for blocking them in the clinic, and presents novel approaches to influence their signaling. The role of IL-12 and IL-23 signaling in the development of IBD is highlighted by the fact that patients with complete loss-of-function mutations in IL23R, IL12RB1, or IL12B do not develop spontaneous inflammatory bowel disease [108]. A recent paper by Ahmed et al. showed that the main cells expressing the IL-23 receptor in the gut are T cells and innate lymphoid group 3 cells (ILC3) [109]. The important involvement of the IL12 and IL23 pathway in the development of IBD is also highlighted in a recent paper by Miyake et al. in which they described the role of the rs6887695 polymorphism of the IL12B gene encoding the p40 subunit in increasing the risk of developing UC [110]. Iliopoulou et al. described the specific role of IL23 in a mouse model of TNF overproduction resembling human CD and found that IL-23 plays a predominant role in the severity of ileitis. Furthermore, histological examination and analysis of immune cell infiltration indicated that the absence of IL-23 leads to a significant reduction in the number of infiltrating immune cells, such as neutrophils and macrophages, and a decrease in the expression of pro-inflammatory cytokines, such as TNF and IFN-γ. They also showed that the main source of IL-23 in the ileum during ileitis is neutrophils, especially their CD14+ subset. The inducer of IL-23 expression by these neutrophils is the environment in the intestine [111]. Furthermore, Jacobse et al. demonstrated the destabilizing effect of IL-23 on Treg lymphocytes in a mouse model of IBD [108]. However, IL-23 may have a protective tissue role in homeostasis or after acute infection. The mechanisms that shape these beneficial versus pathological outcomes are poorly understood. Ahmed et al. showed that IL-23 together with gut microbiota in a FOXO1- and STAT3-dependent manner strongly upregulated the checkpoint molecule of cytotoxic T-lymphocyte-associated antigen-4 (CTLA-4) immunoregulation on ILC3, and that IL-23 induction of CTLA-4 + ILC3 was necessary and sufficient to reduce co-stimulatory molecules and increase PD-L1 bioavailability on myeloid cells. Increased CTLA-4 expression on ILC3 in response to IL23 correlated with immunoregulation in IBD [109]. IL-23, together with IL-22, also plays a key role in the formation of tertiary lymphoid organs in the colon, which may act to improve the mucosal barrier [112]. Due to their important proven role in the pathogenesis of IBD, IL-12 and IL-23 have become clinically relevant targets for the treatment of these diseases [113]. Depending on the chosen target, the drugs developed block the p35 and p40 subunits of IL-12 and the p19 and p40 subunits of IL-23.

Ustekinumab, an IL12/23p40 antagonist, was approved by the Food and Drug Administration for CD treatment in 2016, and clinical data showed that patients experienced symptom relief and achieved clinical remission of IBD [80,113]. In recent years, the focus has been on the clinical aspects of ustekinumab use in the treatment of IBD. In a systematic review and meta-analysis, Honap et al. summarized the efficacy and safety of ustekinumab in the treatment of a total of 4400 patients with CD and UC. For CD, the mean clinical remission rate was 34% after induction and 31% after 1 year of treatment. For UC, the remission rate after induction was 39%. Approximately 5.6% of cases had serious adverse effects. The most common side effects were infections, joint pain, and skin reaction [114]. McDonald et al. demonstrated a relationship between ustekinumab levels and the likelihood of mucosal healing and response in patients with CD. Patients with higher ustekinumab levels (>2.3 µg/mL) were more than 10 times more likely to achieve mucosal healing. Full sensitivity (100%) and high specificity (90.6%) were achieved for this concentration threshold in predicting treatment response [115]. Bertani et al. attempted to identify a biomarker to predict therapeutic response to ustekinumab CD. This pilot study showed that IL-23 and fecal calprotectin could be reliable biomarkers in predicting the therapeutic outcome of ustekinumab therapy in CD. The correlation between baseline serum IL-23 levels and mucosal healing at 48 weeks is particularly strong [116].

In recent years, work to effectively block IL-23 has also included monoclonal antibodies targeting the p19 subunit of IL23. One of these was mirikizumab. These have mainly focused on clinical data, but the molecular basis of the drug has also been described. Most of the newly published clinical data relate to the use of mirikizumab in the treatment of moderate to severe UC. The results of these papers are consistent and confirm the beneficial effect of treatment with this drug in inducing clinical, endoscopic, and histological remission and improving patients’ quality of life. At the same time, no new significant safety concerns have been raised [117,118,119,120]. D’Haens evaluated mirikizumab for the treatment of moderate to severe UC in patients who did not achieve a clinical response after 12 weeks of treatment with the drug. After three additional doses, 53.7% of them achieved a clinical response after 24 weeks. Furthermore, at 52 weeks: 72.2% of patients achieved a sustained clinical response, 43.1% achieved endoscopic remission, and 36.1% achieved clinical remission. The study also showed that patients who lost their response during maintenance therapy were able to achieve a response again with three rescue doses of mirikizumab. In this group, 63.2% regained symptomatic response and 36.8% achieved symptomatic remission. In addition, the study identified potential predictors of response to prolonged induction, such as no prior treatment with biologics or tofacitinib, no use of immunomodulators at the start of treatment, age over 40 years, and improvement in Mayo score after 12 weeks [121]. Steere et al. analyzed the molecular mechanisms of action of mirikizumab in the treatment of moderate to severe UC. The greatest changes in gene expression were observed in the 200 mg mirikizumab group, where they found a reduction in the expression of genes related to inflammation (e.g., IL-1b, MMP1, MMP3, S100A8), and an increase in the expression of genes related to epithelial recovery and function, such as AQP8 (aquaporin-8), ABCG2, HMGCS2. Changes in gene expression correlated with improved clinical outcomes, such as reduced number of bowel movements, reduced urgency to defecate, and improved quality of life for patients. Increased expression of AQP8, which encodes a water transporter, may help reduce the frequency and severity of diarrhea in patients. Changes in the expression of genes associated with treatment resistance, such as OSMR (oncostatin M receptor), suggest that mirikizumab may act on mechanisms of resistance to biologic drugs, including TNF inhibitors, which may be particularly important in treating patients who do not respond to traditional therapies [122]. The safety and efficacy of mirikizumab have also been re-tested in patients with moderate to severe CD. Sands et al. found that at week 12, endoscopic response was significantly higher in patients receiving mirikizumab than placebo. Endoscopic response at week 52 was 58%, and adverse event rates in mirikizumab-treated patients were similar to placebo [123]. Another monoclonal antibody that blocks p19 IL23 is risankizumab. The results of two randomized controlled trials evaluating the efficacy and safety of risankizumab, in patients with moderately to severely active UC, were published in September 2024. Among the 975 patients analyzed in the induction study, clinical remission rates at week 12 were 20.3% after 1200 mg risankizumab and 6.2% after placebo. Among the 548 patients analyzed in the maintenance study, clinical remission rates at week 52 were 40.2% after 180 mg risankizumab, 37.6% after 360 mg risankizumab, and 25.1% after placebo. No new safety risks were detected in the treatment groups. Compared with placebo, risankizumab improved clinical remission rates in the induction study and in the maintenance study in patients with moderately to severely active UC [124]. Risankizumab has also been tested in the treatment of CD. Ferrante et al. evaluated the efficacy of risankizumab as maintenance therapy in patients with a moderate to severe course of this disease. The study included 462 patients who achieved a clinical response after initial treatment with intravenous risankizumab. Patients were randomly assigned to a group receiving risankizumab subcutaneously at doses of 360 mg, 180 mg, or placebo every 8 weeks for 52 weeks. The results were as follows: 68.6% of patients in the 360 mg group and 70.8% in the 180 mg group maintained remission, compared with only 56.3% in the placebo group (*p* < 0.05). In the majority of patients who achieved normal calprotectin and CRP levels during induction therapy, these values were maintained in the risankizumab groups for 52 weeks [125]. Wang et al. found that risankizumab reduced the number of pro-inflammatory cells, with a particularly strong effect on reducing inflammatory monocytes and fibroblasts in patients with IBD [126]. Another monoclonal antibody that blocks IL23p19 is brazikumab. Danese et al. tested it for the treatment of moderate to severe CD in patients who had failed to respond to or were non-tolerant to TNF-α inhibitor therapy. At week 56, 53.8% of patients achieved a clinical response and 46.2% achieved clinical remission, while at week 112, 41.3% of patients maintained clinical response and 36.5% maintained remission. Adverse events occurred in 83.7% of patients, but most were mild or moderate. The most common adverse events included nasopharyngitis, headache, and abdominal pain. No serious adverse events related to opportunistic infections, deaths, or tumors were reported [127]. The next monoclonal antibody targeting IL23p19 is guselkumab, which has been tested in the treatment of both UC and CD. Peyrin-Biroulet et al. evaluated it in the treatment of moderate to severe UC. At week 12 of treatment with guselkumab, a significantly higher proportion of patients achieved a clinical response (at both 200 mg and 400 mg doses) than in the placebo group (61.4% and 60.7% vs. 27.6%, respectively). At the same time, guselkumab was well tolerated, with adverse event rates comparable to placebo. No serious infections or drug-related reactions such as anaphylaxis were reported [128]. Danese et al. found that in patients with moderate to severe CD, guselkumab administered intravenously as induction treatment and subcutaneously as maintenance treatment achieved high clinical and endoscopic success rates up to week 48. They did not identify any new safety risks during the use of this drug [129].

Recently, studies have also yielded papers describing experimental novel approaches to IL-12 and IL-23 for the treatment of IBD (Table 6). Wang et al. developed a novel bispecific nanobody (BsNb) that simultaneously targets TNF-α and IL-23p19. In vivo studies showed that BsNb-Fc was more effective in alleviating the symptoms of colitis in mice induced by DSS and TNBS than the combination of infliximab and ustekinumab. BsNb-Fc reduced levels of pro-inflammatory cytokines, such as TNF-α, IL-17, and IL-23, and reduced infiltration of inflammatory cells in intestinal tissues, including neutrophils and macrophages. Histological examination showed that BsNb-Fc reduced inflammation-induced intestinal damage, which included less ulceration and less destruction of crypts. In vitro: BsNb-Fc inhibited cytokine secretion by macrophages and the proliferation and differentiation of CD4+ T cells into pro-inflammatory Th17 phenotypes, which are involved in the inflammatory response in IBD [130]. Cui et al. described a novel approach to treating CD by using regulatory T cells genetically modified to express a chimeric antigen receptor (CAR) targeting the interleukin 23 receptor (IL23R). This allows CAR to activate Treg cells in response to IL23R in tissues. IL23R-CAR Treg cells were exposed to IL23R in vitro by contacting IL23R-coated beads and CD patient cells. In response to IL23R, these cells activated and effectively suppressed effector T cell proliferation. Transwell assays also demonstrated the ability to suppress the immune response without direct contact with effector T cells, suggesting a potential immunosuppressive effect at a distance. In mouse models, luciferase-labelled IL23R-CAR Treg cells specifically migrated to sites where IL23R expression was elevated (IL23R+), whereas other control Treg did not show a similar ability to migrate autoimmunity and chronic inflammation [131].

### 2.7. Interleukin 33

Interleukin 33 (IL-33) is a newly characterized cytokine, a member of the IL-1 superfamily, which is passively released following cell damage and necrosis [132,133]. Expression of IL-33 and its receptor ST2 has been well established in the gastrointestinal tract, being an important enhancer of innate immunity in the intestinal mucosa [134]. While IL-33 is expressed mainly in mucosa and myofibroblasts, its receptor is expressed mainly on immune cells such as ILC2, Treg, T helper cells, and CD8+ T cells [135]. This allows IL-33/ST2 signaling to act as a bridge between tissue damage and immune system orchestration, which may be a critical component of gut immunity [136]. IL-33 is known to play an important role in the pathogenesis of IBD [137]. Patient biopsy studies have shown an increase in IL-33 levels in patients with IBD, especially UC, compared to healthy controls [138]. The role of IL-33 in the development of IBD is complex. On the one hand, it stimulates UC exacerbations by inducing an IL4-dependent immune response and increases neutrophil invasion in acute inflammation. On the other hand, it regulates microbiota homeostasis, reduces chronic inflammation, accelerates healing, and promotes intestinal reconstitution by polarizing macrophages from M1 to M2 and regenerating cup cells [137]. While the monoclonal antibody blocking the IL-33 receptor ST2 is already being tested in respiratory diseases, it has not yet been evaluated in the treatment of IBD [139]. A recent study outlines the potential use of IL-33 in the clinic (Table 7). In the study by Toskas et al., a significant correlation was found between an increase in IL-33 levels after anti-TNFα and ustekinumab treatment and a reduction in disease activity indices, suggesting that IL-33 may prove to be a good biomarker of treatment efficacy in the future [140].

## 3. Conclusions

Recent studies on the role of interleukins in the pathogenesis of IBD have provided valuable data that may significantly influence future therapeutic strategies (Table 8). Interleukins, which are key mediators of the immune response, play an important role in both the initiation and progression of IBD. Many papers have focused on precisely defining the role of individual interleukins, while providing new clinical data, particularly in the context of interleukin 23 and interleukin 1β blockade. A number of innovative approaches to the use of interleukins in the treatment of IBD have also been described in recent years, opening the way to more targeted and effective therapies. Further research is focusing on better understanding the mechanisms of action of these molecules and identifying biomarkers with prognostic and therapeutic potential. With these discoveries, it becomes possible to develop personalized treatment strategies tailored to the individual immunological profiles of patients. As a result, interleukins are not only becoming a key element in understanding the pathogenesis of IBD, but also a fundament of modern therapies that have the potential to revolutionize the management and treatment of this complex disease.

## Figures and Tables

**Table 1:** Summary of recent studies on IL-1β in IBD.

Study	Conclusions	Ref.
Shaul et al.	Canakinumab used in children with a very early form of inflammatory bowel disease with an autoinflammatory phenotype resulted in significant improvements in clinical symptoms and biochemical markers of the disease, reduced hospitalization rates, and length of hospitalization	[48]
Truyens et al.	Anakinra achieves therapeutic success in a UC patient	[49]
Cai et al.	Molecule 10v blocking IL-1β signaling via the NLRP3 and AIM-2 inflamasome and reducing STAT1 and STAT5 expression in the JAK/STAT pathway achieved better efficacy than sulfasalazine in a mouse model	[50]
Zhu et al.	Activation of the estrogen receptor β (Erβ) by, among other things, reducing IL-1β production results in a reduction of colitis symptom severity in a mouse model in a manner comparable to 5-aminosalicylic acid	[51]

**Table 2:** Summary of recent studies on IL-6 in IBD.

Study	Conclusions	Ref.
Gesiorowski et al.	The IL-6 and TNF-α inhibitor cs130-TNFVHHFc effectively inhibited TNFα-induced apoptosis of L929 cells, and inhibited STAT3 phosphorylation	[62]
Godala et al.	Measurements of IL-6 and IL-1β levels can provide additional information on the nutritional status of IBD patients, especially in the context of body fat and muscle mass	[63]

**Table 3:** Summary of recent studies on IL-10 in IBD.

Study	Conclusions	Ref.
Griffin et al.	Neutralizing anti-IL-10 autoantibodies can cause IBD, resembling IL-10-associated innate immune defects; patients with anti-IL-10 autoantibodies may benefit from therapies targeting the removal of B cells that produce these autoantibodies	[71]
Liu et al.	An increase in IL-10 can be achieved by mesenchymal cells	[72]
Zegarra Ruiz et al.	An increase in IL-10 can be achieved by manipulating the gut microbiota	[73]
Matsui et al.	An increase in IL-10 can be achieved by pharmacological blockade of the KCa3 potassium channel.1	[74]
(#NCT04583358)	Concentrating biologically active IL-10 on the intestinal lamina propria, the AMT-1 molecule is currently in a phase II clinical trial for the treatment of UC (#NCT04583358)	[59,76]
Ben-Khemis et al.	STAT3 phosphorylation induced by IL-10 can be inhibited by TNFα through the NOX2-ROS-Lyn-SHP-1 pathway; SHP-1 inhibitors (such as NSC-87877) can block this mechanism, restoring STAT3 phosphorylation and protection against inflammation	[77]

**Table 4:** Summary of recent studies on IL-17 in IBD.

Study	Conclusions	Ref.
Cai et al.	IL-17B, IL-17E, and IL-17RB are associated with UC, while IL-17C and IL-17RC are associated with CD	[84]

**Table 5:** Summary of recent studies on IL-22 in IBD.

Study	Conclusions	Ref.
He et al.	IL-22 plays a key role in Paneth cell differentiation	[92]
Chen et al.	IL-22 is involved in the function of immunomodulatory mesenchymal stem cells derived from Peyer’s tufts (MSCsPP) capable of alleviating IBD in a mouse model	[93]
Zhu et al.	IL-22.Fc reduces colitis severity, weight loss and increases survival in a mouse model of *Citrobacter rodentium*-induced colitis	[94]
Ninnemann et al.	TNF interferes with the tissue-repair program by inducing soluble natural antagonist IL-22 (IL-22Ra2; IL-22BP) in the colon and abrogates IL-22/STAT3-mediated mucosal repair during colitis	[89]
Fantou et al.	Higher levels of IL-22BP were detected in the ileum than in the colon in both CD patients and controls; the IL-22/IL-22BP ratio, reflecting IL-22 activity, was higher in the colon of CD patients, suggesting greater bioavailability of IL-22 in this location; the main sources of IL-22 BP are eosinophils and mononuclear phagocytes; active smokers had higher levels of IL-22BP in the colon	[95]
Breugelmans et al.	IL-22 induces intestinal barrier dysfunction via the MUC13 protein	[96]
Pavlidis et al.	IL-22 regulates neutrophil recruitment in ulcerative colitis and is associated with resistance to ustekinumab treatment	[97]
Pravoverov et al.	IL-22 increases the expression of oncogenic microtubule-associated serine/threonine kinase (MASTL), possibly promoting its association with carbonic anhydrase IX (CAIX) to promote cell survival and proliferation	[98]
Kuchař et al.	Modulation of the IL-22/IL-22R1 pathway by the antagonistic ABR167 protein reduces IBD severity in a mouse model	[99]

**Table 6:** Summary of recent studies on IL-12 and IL-23 in IBD.

Study	Conclusions	Ref.
Miyake et al.	The rs6887695 polymorphism of the IL12B gene encoding the p40 subunit plays a role in increasing the risk of developing UC	[110]
Iliopoulou et al.	IL-23 plays a predominant role in the severity of ileal inflammation in a mouse model of CD; the absence of IL-23 leads to a significant reduction in the number of infiltrating immune cells such as neutrophils and macrophages and a decrease in the expression of pro-inflammatory cytokines such as TNF and IFN-γ; the main source of IL-23 in the ileum during its inflammation is neutrophils, especially their CD14+ subset; The intestinal environment is an inducer of IL-23 expression by neutrophils	[111]
Jacobse et al.	IL-23 shows destabilizing effects on Treg lymphocytes in a mouse model	[108]
Ahmed et al.	The main cells expressing the IL-23 receptor in the gut are T lymphocytes and innate lymphoid group 3 cells (ILC3). IL-23, together with the gut microbiota, in a FOXO1- and STAT3-dependent manner, strongly upregulates the immunoregulation checkpoint molecule cytotoxic T-lymphocyte-associated antigen-4 (CTLA-4) on ILC3 and IL-23 induction of CTLA-4 + ILC3 was necessary and sufficient to reduce co-stimulatory molecules and increase PD-L1 bioavailability on myeloid cells. Increased CTLA-4 expression on ILC3 in response to IL23 correlated with immunoregulation in IBD	[109]
Gomez-Nguyen et al.	IL-23, together with IL-22, plays a key role in the formation of tertiary lymphoid organs in the colon	[112]
Honap et al.	The use of ustekinumab in the treatment of CD achieved an average clinical remission rate of 34% after induction and 31% after 1 year of treatment, and in the treatment of UC, the remission rate after induction was 39%. Approximately 5.6% of cases had serious adverse effects. The most common side effects were infections, joint pain, and skin reactions	[114]
McDonald et al.	CD patients with higher levels of ustekinumab (>2.3 µg/mL) were more than 10 times more likely to achieve mucosal healing	[115]
Bertani et al.	IL-23 and fecal calprotectin may be reliable biomarkers in predicting therapeutic outcome of ustekinumab therapy in CD	[116]
Sands et al., Magro et al., Kobayashi et al., D’Haens et al.	Mirikizumab achieves induction of clinical, endoscopic, histological remission and improved quality of life in patients with moderate to severe UC	[117,118,119,120]
D’Haens et al.	Additional doses of miricizumab may ensure that a therapeutic response is achieved in patients with moderate to severe UC who have previously failed to achieve a response after 12 weeks of treatment with this drug	[121]
Steere et al.	Mirikizumab for the treatment of moderate to severe UC reduces the expression of genes associated with inflammation (e.g., IL-1b, MMP1, MMP3, S100A8) and increases the expression of genes associated with epithelial restoration and function, such as AQP8 (aquaporin-8), ABCG2, HMGCS2; Changes in gene expression during mirikizumab treatment correlate with improved clinical outcomes	[122]
Sands et al.	Mirikuzmab achieves better clinical response in patients with moderate to severe CD at week 12 of treatment than placebo	[123]
Louis et al.	Risankizumab achieves better clinical response in patients with moderate to severe UC at week 12 of treatment than placebo	[124]
Ferrante et al.	Risankizumab achieves a better clinical response on maintenance therapy in patients with moderate to severe UC than placebo; In the majority of patients who achieved normal calprotectin and CRP levels during induction therapy, these values were maintained in the risankizumab groups for 52 weeks	[125]
Wang et al.	Risankizumab reduces the number of pro-inflammatory cells, mainly monocytes and inflammatory fibroblasts in patients with IBD	[126]
Danese et al.	Brazicumab was tested for the treatment of moderate to severe CD in patients who did not respond to or tolerate TNF-α inhibitor therapy. At week 56, 53.8% of patients achieved clinical response and 46.2% clinical remission, while at week 112, 41.3% of patients maintained clinical response and 36.5% remission. Adverse events occurred in 83.7% of patients, but most were of mild to moderate severity	[127]
Peyrin-Biroulet et al.	In the treatment of moderate to severe UC at week 12, guselkumab achieved a significantly higher proportion of patients achieving a clinical response than in the placebo group. At the same time, guselkumab was well tolerated, with adverse event rates comparable to placebo	[128]
Danese et al.	In patients with moderate to severe CD, guselkumab administered intravenously as induction treatment and subcutaneously as maintenance treatment achieved high clinical and endoscopic efficacy rates up to week 48	[129].
Wang et al.	Bispecific nanobodies (BsNb) simultaneously targeting TNF-α and IL-23p19 more effectively than the combination of infliximab and ustekinumab relieve colitis symptoms in a mouse model	[130]
Cui et al.	Regulatory T cells genetically modified to express a chimeric antigen receptor (CAR) targeting interleukin 23 receptor (IL23R): (1) in response to IL23R, they activated and effectively suppressed effector T cell proliferation, (2) in mouse models of IL23R-CAR Treg cells, migrated specifically to sites where IL23R expression was elevated (IL23R+)	[131]

**Table 7:** Summary of recent studies on IL-33 in IBD.

Study	Conclusions	Ref.
Toskas et al.	There is a correlation between an increase in IL-33 levels after anti-TNFα and ustekinumab treatment and a reduction in disease activity indices, suggesting that IL-33 may prove to be a good biomarker of treatment efficacy in the future	[140]

**Table 8:** Summary of recent studies on the interleukins discussed in the article in IBD.

Interleukin	Study	Conclusions	Ref.
IL-1β	Shaul et al.	Canakinumab used in children with a very early form of inflammatory bowel disease with an autoinflammatory phenotype resulted in significant improvements in clinical symptoms and biochemical markers of the disease, reduced hospitalization rates, and lengths of hospitalization	[48]
IL-1β	Cai et al.	Molecule 10v blocking IL-1β signaling via the NLRP3 and AIM-2 inflamasome and reducing STAT1 and STAT5 expression in the JAK/STAT pathway achieved better efficacy than sulfasalazine in a mouse model	[50]
IL-1β	Zhu et al.	Activation of the estrogen receptor β (Erβ) by, among other things, reducing IL-1β production results in a reduction of colitis symptom severity in a mouse model in a manner comparable to 5-aminosalicylic acid	[51]
IL-6	Gesiorowski et al.	The IL6 and TNF-α inhibitor cs130-TNFVHHFc effectively inhibited TNFα-induced apoptosis of L929 cells, and inhibited STAT3 phosphorylation	[62]
IL-6	Godala et al.	Measurements of IL-6 and IL-1β levels can provide additional information on the nutritional status of IBD patients, especially in the context of body fat and muscle mass	[63]
IL-10	Griffin et al.	Neutralizing anti-IL-10 autoantibodies can cause IBD, resembling IL-10-associated innate immune defects; patients with anti-IL-10 autoantibodies may benefit from therapies targeting the removal of B cells that produce these autoantibodies	[71]
IL-10	Liu et al.	An increase in IL-10 can be achieved by mesenchymal cells	[72]
IL-10	Zegarra Ruiz et al.	An increase in IL-10 can be achieved by manipulating the gut microbiota	[73]
IL-10	Matsui et al.	An increase in IL-10 can be achieved by pharmacological blockade of the KCa3 potassium channel.1	[74]
IL-10	(#NCT04583358)	Concentrating biologically active IL-10 on the intestinal lamina propria, the AMT-1 molecule is currently in a phase II clinical trial for the treatment of UC (#NCT04583358)	[76]
IL-10	Ben-Khemis et al.	STAT3 phosphorylation induced by IL-10 can be inhibited by TNFα through the NOX2-ROS-Lyn-SHP-1 pathway; SHP-1 inhibitors (such as NSC-87877) can block this mechanism, restoring STAT3 phosphorylation and protection against inflammation	[77]
IL-17	Cai et al.	IL-17B, IL-17E, and IL-17RB are associated with UC, while IL-17C and IL-17RC are associated with CD	[84]
IL-22	He et al.	IL-22 plays a key role in Paneth cell differentiation	[92]
IL-22	Chen et al.	IL-22 is involved in the function of immunomodulatory mesenchymal stem cells derived from Peyer’s tufts (MSCsPP) capable of alleviating IBD in a mouse model	[93]
IL-22	Zhu et al.	IL-22.Fc reduces colitis severity, weight loss and increases survival in a mouse model of *Citrobacter rodentium*-induced colitis	[94]
IL-22	Ninnemann et al.	TNF interferes with the tissue repair program by inducing soluble natural antagonist IL-22 (IL-22Ra2; IL-22BP) in the colon and abrogates IL-22/STAT3-mediated mucosal repair during colitis	[89]
IL-22	Fantou et al.	Higher levels of IL-22BP were detected in the ileum than in the colon in both CD patients and controls; the IL-22/IL-22BP ratio, reflecting IL-22 activity, was higher in the colon of CD patients, suggesting greater bioavailability of IL-22 in this location; the main sources of IL-22 BP are eosinophils and mononuclear phagocytes; active smokers had higher levels of IL-22BP in the colon	[95]
IL-22	Breugelmans et al.	IL-22 induces intestinal barrier dysfunction via the MUC13 protein	[96]
IL-22	Pavlidis et al.	IL-22 regulates neutrophil recruitment in ulcerative colitis and is associated with resistance to ustekinumab treatment	[97]
IL-22	Pravoverov et al.	L-22 increases the expression of oncogenic microtubule-associated serine/threonine kinase (MASTL), possibly promoting its association with carbonic anhydrase IX (CAIX) to promote cell survival and proliferation	[98]
IL-22	Kuchař et al.	Modulation of the IL-22/IL-22R1 pathway by the antagonistic ABR167 protein reduces IBD severity in a mouse model	[99]
IL-12, IL-23	Miyake et al.	The rs6887695 polymorphism of the IL12B gene encoding the p40 subunit plays a role in increasing the risk of developing UC	[110]
IL-12, IL-23	Iliopoulou et al.	IL-23 plays a predominant role in the severity of ileal inflammation in a mouse model of CD; the absence of IL-23 leads to a significant reduction in the number of infiltrating immune cells such as neutrophils and macrophages and a decrease in the expression of pro-inflammatory cytokines such as TNF and IFN-γ; the main source of IL-23 in the ileum during its inflammation is neutrophils, especially their CD14+ subset; The intestinal environment is an inducer of IL-23 expression by neutrophils	[111]
IL-12, IL-23	Jacobse et al.	IL-23 shows destabilizing effects on Treg lymphocytes in a mouse model	[108]
IL-12, IL-23	Ahmed et al.	The main cells expressing the IL-23 receptor in the gut are T lymphocytes and innate lymphoid group 3 cells (ILC3). IL-23, together with the gut microbiota, in a FOXO1- and STAT3-dependent manner, strongly upregulates the immunoregulation checkpoint molecule cytotoxic T-lymphocyte-associated antigen-4 (CTLA-4) on ILC3 and IL-23 induction of CTLA-4 + ILC3 was necessary and sufficient to reduce co-stimulatory molecules and increase PD-L1 bioavailability on myeloid cells. Increased CTLA-4 expression on ILC3 in response to IL23 correlated with immunoregulation in IBD	[109]
IL-12, IL-23	Gomez-Nguyen et al.	IL-23, together with IL-22, plays a key role in the formation of tertiary lymphoid organs in the colon	[112]
IL-12, IL-23	Honap et al.	The use of ustekinumab in the treatment of CD achieved an average clinical remission rate of 34% after induction and 31% after 1 year of treatment, and in the treatment of UC, the remission rate after induction was 39%. Approximately 5.6% of cases had serious adverse effects. The most common side effects were infections, joint pain, and skin reactions	[114]
IL-12, IL-23	McDonald et al.	CD patients with higher levels of ustekinumab (>2.3 µg/mL) were more than 10 times more likely to achieve mucosal healing	[115]
IL-12, IL-23	Bertani et al.	IL-23 and fecal calprotectin may be reliable biomarkers in predicting therapeutic outcome of ustekinumab therapy in CD	[116]
IL-12, IL-23	Sands et al., Magro et al., Kobayashi et al., D’Haens et al.	Mirikizumab achieves induction of clinical, endoscopic, histological remission and improved quality of life in patients with moderate to severe UC	[117,118,119,120]
IL-12, IL-23	D’Haens et al.	Additional doses of miricizumab may ensure that a therapeutic response is achieved in patients with moderate to severe UC who have previously failed to achieve a response after 12 weeks of treatment with this drug	[121]
IL-12, IL-23	Steere et al.	Mirikizumab for the treatment of moderate to severe UC reduces the expression of genes associated with inflammation (e.g., IL-1b, MMP1, MMP3, S100A8) and increases the expression of genes associated with epithelial restoration and function, such as AQP8 (aquaporin-8), ABCG2, HMGCS2; Changes in gene expression during mirikizumab treatment correlate with improved clinical outcomes	[122]
IL-12, IL-23	Sands et al.	Mirikuzmab achieves better clinical response in patients with moderate to severe CD at week 12 of treatment than placebo	[123]
IL-12, IL-23	Louis et al.	Risankizumab achieves better clinical response in patients with moderate to severe UC at week 12 of treatment than placebo	[124]
IL-12, IL-23	Ferrante et al.	Risankizumab achieves a better clinical response on maintenance therapy in patients with moderate to severe UC than placebo; In the majority of patients who achieved normal calprotectin and CRP levels during induction therapy, these values were maintained in the risankizumab groups for 52 weeks	[125]
IL-12, IL-23	Wang et al.	Risankizumab reduces the number of pro-inflammatory cells, mainly monocytes and inflammatory fibroblasts in patients with IBD	[126]
IL-12, IL-23	Danese et al.	Brazicumab was tested for the treatment of moderate to severe CD in patients who did not respond to or tolerate TNF-α inhibitor therapy. At week 56, 53.8% of patients achieved clinical response and 46.2% clinical remission, while at week 112, 41.3% of patients maintained clinical response and 36.5% remission. Adverse events occurred in 83.7% of patients, but most were of mild to moderate severity	[127]
IL-12, IL-23	Peyrin-Biroulet et al.	In the treatment of moderate to severe UC at week 12, guselkumab achieved a significantly higher proportion of patients achieving a clinical response than in the placebo group. At the same time, guselkumab was well tolerated, with adverse event rates comparable to placebo	[128]
IL-12, IL-23	Danese et al.	In patients with moderate to severe CD, guselkumab administered intravenously as induction treatment and subcutaneously as maintenance treatment achieved high clinical and endoscopic efficacy rates up to week 48	[129]
IL-12, IL-23	Wang et al.	Bispecific nanobodies (BsNb) simultaneously targeting TNF-α and IL-23p19 more effectively than the combination of infliximab and ustekinumab relieve colitis symptoms in a mouse model	[130]
IL-12, IL-23	Cui et al.	Regulatory T cells genetically modified to express a chimeric antigen receptor (CAR) targeting interleukin 23 receptor (IL23R): (1) in response to IL23R, they activated and effectively suppressed effector T cell proliferation, (2) in mouse models of IL23R-CAR Treg cells, migrated specifically to sites where IL23R expression was elevated (IL23R+)	[131]
IL-33	Toskas et al.	There is a correlation between an increase in IL-33 levels after anti-TNFα and ustekinumab treatment and a reduction in disease activity indices, suggesting that IL-33 may prove to be a good biomarker of treatment efficacy in the future	[140]
